# “Sexual” Population Structure and Genetics of the Malaria Agent *P. falciparum*

**DOI:** 10.1371/journal.pone.0000613

**Published:** 2007-07-18

**Authors:** Themba Mzilahowa, Philip J. McCall, Ian M. Hastings

**Affiliations:** 1 Liverpool School of Tropical Medicine, Liverpool, United Kingdom; 2 Malawi-Liverpool Wellcome Trust Clinical Research Programme, Blantyre, Malawi; Mahidol University, Thailand

## Abstract

The population genetics and structure of *P. falciparum* determine the rate at which malaria evolves in response to interventions such as drugs and vaccines. This has been the source of considerable recent controversy, but here we demonstrate the organism to be essentially sexual, in an area of moderately high transmission in the Lower Shire Valley, Malawi. Seven thousand mosquitoes were collected and dissected, and genetic data were obtained on 190 oocysts from 56 infected midguts. The oocysts were genotyped at three microsatellite loci and the MSP1 locus. Selfing rate was estimated as 50% and there was significant genotypic linkage disequilibrium (LD) in the pooled oocysts. A more appropriate analysis searching for genotypic LD in outcrossed oocysts and/or haplotypic LD in the selfed oocysts found no evidence for LD, indicating that the population was effectively sexual. Inbreeding estimates at MSP1 were higher than at the microsatellites, possibly indicative of immune action against MSP1, but the effect was confounded by the probable presence of null mutations. Mating appeared to occur at random in mosquitoes and evidence regarding whether malaria clones in the same host were related (presumably through simultaneous inoculation in the same mosquito bite) was ambiguous. This is the most detailed genetic analysis yet of *P. falciparum* sexual stages, and shows *P. falciparum* to be a sexual organism whose genomes are in linkage equilibrium, which acts to slow the emergence of drug resistance and vaccine insensitivity, extending the likely useful therapeutic lifespan of drugs and vaccines.

## Introduction

Malaria caused by *Plasmodium falciparum* is a major public health problem in many countries worldwide, particularly in sub-Saharan Africa. The genetic structure of *P. falciparum* populations determines how the species evolves in response to external pressures such as novel drug and vaccine deployment, and has been a long-standing topic of intense debate: some researchers argue that *P. falciparum* is effectively an asexual, clonal organism with long-lasting, stable genotypes (e.g. [1]–[4] and references therein) while others believe it to be an essentially sexual organism with some (transitory) self-fertilisation and a freely recombining genome (e.g. [5]–[13] and references therein).

The debate centres around the consequences of the obligate sexual phase of the parasite lifecycle*. Plasmodium* spp. are haploid in both human and mosquito hosts except for a brief stage in the mosquito midgut where two haploid malaria gametes present in the mosquito's blood meal fuse to form a diploid zygote. This zygote immediately undergoes meiosis to generate four haploid cells that continue in the haploid form, reproducing through mitosis, until several thousand are present in a small sac, the oocyst, attached to the gut wall. Importantly the oocyst contains sufficient DNA that the diploid genotype can be determined by PCR [14]. Genetically-distinct malaria clones are often present in the same human blood meal so this sexual phase provides an opportunity for genetic recombination to occur between genetically-distinct malaria parasites, resulting in the generation of genetic diversity. Effective recombination, or ‘outcrossing’, occurs between genetically-distinct clones and results in the production of novel, recombinant genotypes. Conversely, ‘selfing’ occurs when fertilisation is between genetically-identical gametes and does not result in genetic recombination. Selfing must occur when only one parasite genotype is present in human blood and will also occur by chance even if two or more genotypes are present. Outcrossing has clearly been demonstrated in *P. falciparum* by experimental crosses in chimpanzees [15] and by direct PCR amplification of oocysts [2], [14], [16]. It can also be inferred by a decrease in linkage disequilibrium with increasing genetic map distance [7]. Herein we present data obtained from direct genotyping of oocysts confirming that *P. falciparum* is clearly a sexual organism in an area of moderate to high transmission of sub-Saharan Africa, a region that bears the brunt of malaria mortality.

## Materials and Methods

Malaria infection occurs all year round in the Lower Shire Valley, Malawi, where transmission is seasonal with an estimated malaria inoculation rate of 183 infective bites per person per year [17].

### Mosquito collection and processing

Naturally infected mosquitoes were collected from Chipula [15°98′S, 34°78′E] and Kela [16°2′S, 34°50′E] villages (and occasionally from nearby Belo and Tomali villages) in the lower Shire Valley, southern Malawi. A series of weekly collections were made over a period of 52 weeks (January 2002–January 2003). Each collection was carried out in 3 houses and on each visit a different set of houses was sampled by harvesting adult female anophelines resting within the house by pyrethrum knockdown catch. This kills the mosquitoes so it was not possible to hold them for a period to allow the oocysts to develop and, consequently, this approach results in the harvesting of smaller oocysts and hence less DNA.

Permission to work in the villages was first sought from the village chiefs and householders. Permission to enter the house was sought and the right to refuse or withdraw at any time was respected. Where permission to enter a house was refused, an alternative house in the immediate proximity was selected. Collection of mosquitoes was carried out between 6:00 a.m. and 8:00 a.m. Preparation of houses and mosquito collection followed standard procedures [18], [19]. The owners were requested to refrain from sweeping until after collection, and all food items, cooking utensils and water were moved outside during the exercise. White sheets were laid in the rooms to cover the floors, beds and other flat surfaces. The interior space of the house was then sprayed with a pyrethroid-based household insecticide aerosol, DOOM^®^ (Dichlorovos, Tetramethrin and d-Phenothrin; Robertsons Homecare Ltd, South Africa). The eaves and windows were sprayed simultaneously on the outside. The house was exited and doors closed. The sheets were removed for inspection after 10–15 minutes.

All mosquitoes were collected and placed in petri-dishes lined with a damp filter paper. Mosquito samples from the 3 houses were kept in separate petri-dishes and labelled accordingly. Petri-dishes were put in a cooler box and transported to the laboratory in Blantyre for processing. All anophelines were identified fresh using morphological keys [20]. Abdomens of mosquitoes were then removed and dissected to examine midguts for oocysts. Heads and thoraces were put in 1.5 µl eppendorf tubes and stored over silica gel for later DNA extraction. Abdomens were dissected and midguts inspected for the presence of oocysts under a dissecting microscope. The number of oocysts observed on each midgut was recorded. Infected midguts were then transferred into an eppendorf tube containing 96% alcohol [21] for storage.

Individual oocysts were isolated from midguts following methods described by Anthony *et al* [21], with some slight modifications. Firstly, midguts were rehydrated in a 1:1 ethanol:TE buffer solution for 30 minutes in separate eppendorf tubes. An individual midgut was then placed in an agarose-based watchglass and the dissections carried out under a dissecting microscope. Individual oocysts were then teased off the basal lamina of the midgut epithelium using minuten pins mounted on metal rods. Each oocyst was then transferred to a new 1.5 ml eppendorf tube using a 2 µl pipette. Dissecting pins were flame sterilized between each oocyst and pipette tips changed to avoid cross-contamination of DNA.

### Genotyping

Extraction of genomic DNA from individual oocysts was carried out using the QIAGEN Kit following the manufacturer's guidelines for purification of genomic DNA from tissues (QIAamp® DNA Micro Handbook). DNA was eluted in 15 µl of sterile double distilled water.

DNA samples were tested to confirm they were *P. falciparum* [22] and only positive samples were then subsequently genotyped.

*P. falciparum* single oocysts populations were genotyped at 3 microsatellite loci: TA109, TA1&ARA2. This choice was based on the fact that they had previously been shown to be polymorphic [23] , had already been amplified from oocysts [21], and because they are located on 2 separate chromosomes. Each of the microsatellite loci is a single copy in the haploid *P. falciparum* genome and all are trinucleotides. The names, chromosomal location, primer sequences and the dye used are shown in Table 1.

**Table 1:** Primer sequences of the three microsatellite markers used, their chromosome locations and the dye used in electrophoresis.

Locus	Chromosome Location	Sequence	Label
ARA2	11	F 5′ GTA CAT ATG AAT CAC CAA 3′	TET (blue)
		R 5′ GCT TTG AGT ATT ATT AAT A 3′	
		F 5′ GAA TAA ACA AAG TAT TGC T 3′	
TA1	6	F 5′ CTA CAT GCC TAA TGA GCA 3′	FAM (green)
		R 5′ TTT TAT CTT CAT CCC CAC 3′	
		F 5′ CCG TCA TAA GTG CAG AGC 3′	
TAA109	6	F 5′ TAG GGA ACA TCA TAA GGA T 3′	HEX (yellow)
		R 5′ CCT ATA CCA AAC ATG CTA AA 3′	
		F 5′ GGT TAA ATC AGG ACA ACA T 3′	

The primer sequences and amplification conditions were as those described by Anderson *et al*. [5]. In nest 1 PCR amplifications, first and second listed primers were used in a 12 µl reaction (pure Taq Ready-To-Go PCR bead-Amersham Biosciences) [21]. This required an addition of 0.5 µl of template DNA. In nest 2 amplifications, second and third listed (that is internal to the first two and end-labelled) primers were used in a 15 µl reaction. The reaction contained 1 µl of product from primary reaction, 2.5 mM MgCl_2_ (2.5 µl 10×buffer containing 15 mM magnesium chloride), 0.2 units of DNA Polymerase Taq from QIAGEN and 2 pM of each primer.

The cycling conditions for all loci were as follows:

Primary reaction: 2 min, 94°C; (30 sec, 94°C; 30 sec, 42°C; 30 sec, 40°C; 40 sec, 65°C)×25; 2 min, 65°C.

Secondary reaction: 2 min, 94°C; (20 sec, 94°C; 20 sec, 45°C; 30 sec, 65°C)×25; 2 min, 65°C. All reactions were carried out using the GeneAmp® PCR System 2700 (Applied Biosystems, UK).

Alleles at all loci were identified by sizing of PCR products electrophoresed on a Perkin Elmer ABI Prism™ 377 DNA Sequencer, using Genescan and Genotyper software (Applied Biosystems, UK). We discarded data from samples that amplified poorly for particular loci (maximum peak height <200 fluorescent units). Heterozygous alleles were scored if minor peaks were >25% of the height of the predominant allele present.

The primers and cycling conditions for genotyping *MSP1* alleles of block 2 were those given by Snounou *et al.* [24] with a few modifications mainly in the primary reaction (nest 1) as described below Amplifications were performed on a GeneAmp® PCR System 2700 (Applied Biosystems, UK). In the first reaction, oligonucleotide primer pairs corresponding to conserved sequences bracketing the polymorphic regions of the three alleles were included. We used half pure Taq Ready-To-Go PCR beads (Amersham Biosciences) for the primary reaction [21]. This required an addition of 0.5 µl of template DNA. The product generated in the first reaction was then used as a template in 6 separate second nested reactions with specific primer pairs to determine the presence of allelic variants from the MAD20, K1 and RO33 families of the MSP1 block 2. The specificity of the PCR was tested using the *P. falciparum* 3D7 laboratory line as a positive control and double distilled sterile water for a negative control. The oocysts that failed to amplify were re-run up to three times, and if they did not amplify then they were recorded as failed. All the nested reactions were performed in 20 µl and the reaction conditions required the addition of 1.5 mM MgCl_2_, 125 µM of each of the 4 dNTPs, 125 nM final concentration of the oligonucleotide primers and 0.6 units of DNA Taq polymerase. We used the standard 10× PCR buffer containing 15 mM MgCl_2_. The cycling conditions for all loci were as follows. Primary reaction: 5 min, 95°C; (1 min, 94°C; 2 min, 61°C; 2 min, 72°C)×25; 5 min, 72°C. Secondary reaction: 5 min, 95°C; (1 min, 94°C; 2 min, 61°C; 2 min, 72°C)×30; 5 min, 72°C. The final PCR product was analyzed by agarose gel electrophoresis in 1×TBE buffer. 2 µl of the blue loading dye was added to a total of 12 µl of the PCR product and then loaded on a 2% agarose gel. DNA bands were visualized by ultraviolet transillumination after staining with ethidium bromide and digital photographs were taken. Digital photos were then analyzed physically. Molecular masses were assigned using Gene-ruler 100 bp DNA ladder (Promega Corporation). Peaks were selected in following size ranges, K1-like alleles: (160–300 bp), MAD20-like alleles: (125–200) and RO33-like alleles: (175), according to the guidelines for fragment sizes using these primers [24].

### Genetic analysis

The rate of selfing in the *P. falciparum* population was calculated by assuming that oocysts that are homozygous at all four loci are the result of selfing (*i.e.* the products of mating between two genetically-identical parasites). Oocysts that are heterozygous at one or more loci are obviously the products of outcrossing (*i.e.* the products of mating between two genetically-distinct parasites). It is possible that some outcrossed oocysts are homozygous at all loci purely by chance, and hence misclassified as selfed, but this possibility is small because of the extent of genetic diversity at the microsatellite loci. The probability that an outcrossed oocyst would be homozygous at all four loci purely by chance was calculated from allele frequencies, and subtracted from the lower confidence limit of the selfing rate. Selfing rates averaged over all 56 mosquitoes provided a mean selfing rate, with appropriate confidence intervals (CI). As a concrete example, if a mosquito had 2 selfed and one outcrossed oocysts, its selfing rate was 2/3 = 0.666.

**Figure 1:**
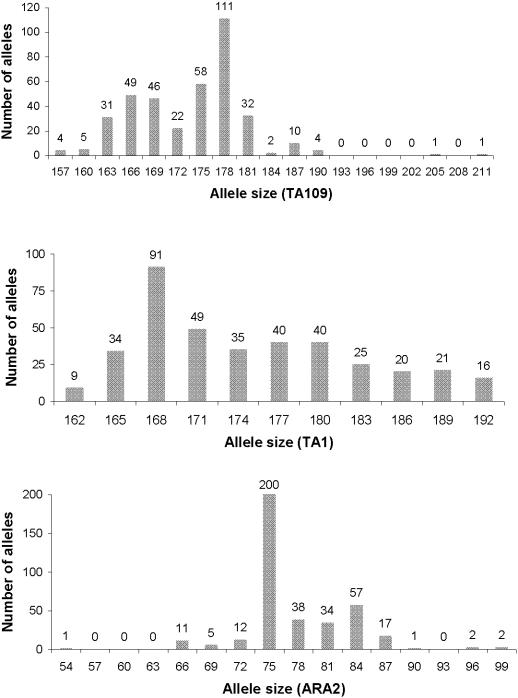
Allele size and numbers collected for the 3 microsatellite markers (TA109, TA1&ARA2) used to study the mating structure of *P. falciparum* populations.

Conventional F statistics [25] were calculated to quantify how mating in the *P. falciparum* population was restricted through its sub-division in separate hosts (*F_st_*), to detect non-random mating of gametes within bloodmeals (*F_is_*), and to obtain an overall level of inbreeding (*F_it_*); their values were obtained using the computer program FSTAT 2.9.3 [26], [27]. It is not sufficient simply to compare *F_st_* values and CI when testing for significant differences in *F_st_* values between *MSP1* and a neutral microsatellite locus [28], because the measures are not independent (oocysts derived from older, more immune people may have increased *F_st_* at both loci). The ratio of *F_st_* was calculated for 5,000 bootstrap replicates, and used to obtain the mean and CI for the ratio [28]. The *TA1* locus was selected for comparison with *MSP1* because it amplified in all oocysts in the data set, and had an even distribution of allele lengths (Figure 1) allowing it to be readily binned into three groups as follows: allele 1 (length ≤168; 35% of total), allele 2 (length 171 to 177; 33% of total), allele 3 (≥180; 32% of total). Having three allelic types has the practical advantage that it can be processed by the computer programme in exactly the same way as the *MSP1* genotypes (which also has 3 alleles), minimising the threat of inadvertent errors or biases.

Linkage disequilibrium (LD) between the four loci was investigated by analysing selfed and outcrossed oocysts (as defined above) separately for reasons that will be discussed later. Genotypic LD in outcrossed oocysts was obtained using FSTAT 2.9.3 [26], [27]. Selfed oocysts reflect the (identical) haplotypes that mated to form the oocyst, so their haplotypes were analysed for haplotypic (*i.e.* gametic) LD. There were only one or at most two selfed oocysts in each mosquito and this high level of population sub-division prevented any effective simultaneous analysis of the whole dataset. The strategy employed was to randomly select one haplotype from each mosquito. As this was a representative sample of haplotypes in the whole *P. falciparum* population, it could be analysed as a single population. One hundred such samples were drawn and investigated for haplotypic LD using the Arlequin v 2.000 [29] and LIAN v3.5 [30] computer programmes, using 100,000 iterations of the latter to obtain the standardised index of association, I_AS_, [31], [32] and its significance. For purposes of illustration, the familiar standardised haplotypic LD statistic for the 2 allele case were obtained by pooling the alleles at each microsatellite locus into two classes of approximately equal frequency among the selfed oocysts, TA109 (≤175 and >175), TA1 (≤171 and >171) ARA2 (≤75 and >75), enabling LD statistics to be obtained by the usual formula (*f_ij_-f_i_f_j_*)/*n*, using Arlequin software.

To investigate whether clones were related within humans (most probably by inoculation in the same mosquito bite) one outcrossed oocyst was randomly selected from each of the 40 midguts with outcrossed oocysts. The resulting dataset was examined for Hardy-Weinberg equilibrium (HWE) and this was repeated 100 times using different random selections. The reasoning is that each outcrossed oocyst will contain two haplotypes sampled at random from a human. If the clones are related, there should be an excess of homozygotes and departure from HWE [33], [34].

## Results

A total of 565 individual oocysts were isolated from 100 infected mosquitoes. A substantial number of oocysts (approximately 146) were lost in the course of dissection because the small size and close proximity of some oocysts made reliable separation and isolation difficult. Eventually 377 (67%) oocysts were tested for the presence of *Plasmodium sp.* parasites of which 52.3% (n = 197) were successfully typed to *Plasmodium* species level. The other 47.7% failed to amplify, possibly because insufficient DNA was present. Two malaria parasites were identified: 97.0% (n = 191) of the oocysts carried *P. falciparum* parasites only, 2.0% (n = 4) were *P. malariae* and a further 1.0% (n = 2) were of each parasite species but co-infecting the same mosquito.

A total of 190 individual *P. falciparum* oocysts from 56 mosquito midguts were genotyped at the four loci. All oocysts successfully amplified at *TA1* and *ARA2* loci, two oocysts failed to amplify at locus *TA109* and 85 (45%) failed to amplify at *MSP1*. Figure 1 shows the distribution of alleles at each of the microsatellite loci: all were highly polymorphic with total numbers of alleles per locus varying from 11 (TA1) to 14 (TA109). At each locus, there was a single dominant allele with an allele frequency of 10% (*TA109*, allele 178), 25% (*TA1*, allele 168) and 50% (*ARA2*, allele 75). The chance of two haplotypes being identical in state purely by chance is a maximum of 0.1×0.25×0.5 = 0.0125 assuming linkage disequilibrium between the loci (see later) and that separate clones in a human (and hence in the bloodmeal of mosquitoes) were derived from separate bites *i.e*. a human could not be infected by two genetically-related clones inoculated in the same bite. To account for this probability of identity in state, the probability was subtracted from the lower limit of CI of the selfing rate (see below). There were 89 ‘selfed’ oocysts (defined as having identical genotypes at all 4 loci) spread over 32 mosquito midguts and 101 outcrossed oocysts spread over 40 midguts.

Selfing rates, averaged over mosquitoes was estimated as 0.44 with confidence limits, adjusted to account for the chance that an oocyst was selfed purely by chance of 0.31–0.56. The *F* statistics are given in Table 2. *F_st_* in MSP1 analysed as putative antigenic class was higher than for TA1, but the difference was not significant under the bootstrapping analysis.

**Table 2:** Sources of inbreeding for *TA109, TA1, ARA2* with (s.e.) and for these three microsatellite combined. The *MSP1* locus is analysed as distinct alleles (distinguished by type and repeat number) and as classes (distinguished only by type of repeat i.e. mad20, k1 or ro33 type)

Locus	*F_it_*	*F_st_*	*F_is_*
*TA109*	0.69 (0.06)	0.43 (0.08)	0.46 (0.08)
*TA1*	0.58 (0.07)	0.54 (0.08)	0.09 (0.07)
*ARA2*	0.35 (0.08)	0.37 (0.08)	−0.03 (0.06)
Combined	0.56	0.45	0.18
*MSP1 (alleles)*	0.62 (0.09)	0.36 (0.07)	0.40 (0.11)
*MSP1 (class)*	0.62 (0.08)	0.49 (0.10)	0.27 (0.12)

There was no indication of linkage disequilibrium (LD) in the dataset. Genotypic LD among outcrossed oocysts was non-significant (p>0.05) as was haplotypic LD among selfed oocysts (I_AS_ = 0.01, p≈0.3). Haplotypic LD among selfed oocysts was −0.36 (TA109 vs. TA1; p≈0.1), −0.14 (TA109 vs. ARA2; p≈0.6) and −0.18 (TA1 vs. ARA2; p≈0.3), the negative coefficients indicating a tendency for shorter alleles at one locus to be associated with longer alleles at the other.

The microsatellite loci generate qualitatively different results in analysis for Hardy-Weinberg equilibrium (HWE). TA109 exhibited homozygous excess and was not in HWE (p = 0.000 for all 100 replicates). TA1 also exhibited homozygous excess and was not in HWE (mean *p* = 0.015 over100 replicates; range 0.000 to 0.15) ARA2 showed no sign of homozygous excess and was in HWE (mean *p* = 0.29 over 100 replicates; range 0.05 to 0.7)

## Discussion

This analysis used material from wild mosquitoes collected by knock-down catches [17] and, consequently, oocysts will be in many different stages of development, because they usually take around 8 days following an infective blood meal to reach their maximum, mature size. Consequently many small, immature oocysts were collected and the low amount of *Plasmodium* DNA in these oocysts is the most likely reason why many failed to amplify even in the initial assays designed to identify *Plasmodium* species. The large number of failures to amplify at the *MSP1* locus may reflect reduced sensitivity of this assay compared to the microsatellite loci, the vast majority of which did amplify, although the presence of ‘null alleles’ (see below) at *MSP1* cannot be discounted and will be discussed later. The microsatellite loci were highly polymorphic in length (Figure 1) confirming their utility in this type of study as a putative-neutral marker to investigate malaria population genetics.

A major potential problem in this type of analysis is the presence of ‘null’ alleles. These alleles do not amplify properly under the PCR conditions and remain undetected. Consequently heterozygous oocysts of type ‘A/null’ will be mis-scored as ‘AA’ homozygotes; this results in overestimates of selfing rates and F statistics, and causes departure from Hardy-Weinberg equilibrium through (apparent) homozygous excess. Nulls can arise for two reasons. The first, which we will term a ‘molecular null’ is where the null allele contains a mutation in the DNA sequence that prevents the primer binding to it; this is the usual type of null encountered in population genetic studies. The second type, which we will call a ‘functional null’ is more specific to our analysis of *Plasmodium* oocysts. The diploid stage of the zygote is believed to last only a single cell division; the two gametes fuse to make a diploid cell which at its next division splits into 4 haploid meiotic products. These four cells then increase in number through mitosis until the mature oocyst contains several thousand copies of the cells. However, if one of the four meiotic products dies, or contains mutations that slow its growth, it will result in an heterozygous oocyst containing much less than 50% copy number of one of the alleles; PCR amplification may then fail to detect this minority allele and the heterozygous oocyst will be incorrectly scored as homozygous (under our scoring system oocysts were only regarded as heterozygous if a second peak was >25% the height of the predominant). The impact of ‘functional nulls’ may be important when, as in this case, many of the oocysts are immature, contain little DNA, and the assays are operating near the limits of their sensitivity. The potential impact of nulls permeates much of the subsequent interpretation of the data and will be discussed at the relevant points below.

The direct inferred selfing rate (i.e. ‘selfing’ defined as identity at all 4 loci) of 0.44 (95%CI 0.31 to 0.56) is roughly similar to its equivalent F statistic, *F_it_*, of around 0.6 suggesting both methods are robust These results are consistent with three previous estimates obtained from direct genotyping of oocysts. Hill *et al* [35] estimated *F_it_* as 0.33 from Tanzania where malaria inoculation rates are very high (estimated at 64–360 infective bites/person/year) and people frequently harbour multiple infections [36], [37]. In Papua New Guinea which is characterised by relatively low inoculation rates [38] and fewer individuals have multiple infections, a value of *F_it_* = 0.92 was reported [16] although revised calculations allowing for the presence of nulls re-estimated it as 0.48 [39]. Finally, Razakandrainibe *et al* [2] reported *F_it_*≈0.4 averaged over a high transmission area of Western Kenya. All analyses therefore support the view that *P. falciparum* is an effectively sexual organism generating frequent sexual recombination in its genome.

Razakandrainibe *et al* [2] reported a selfing rate obtained using the formula 2*F_is_*/(1+*F_is_*). This is the standard formula for the classical mixed mating model where diploids can self- or cross-fertilise but it is not appropriate in malaria where ‘selfing’ occurs between haploid genotypes. Selfing at the diploid stage reduces genetic variation by 50% at each selfing event: for example if a plant is genotype *Aa* at a locus then self fertilisation produces offspring of genotype *AA, Aa aA* or *aa* and the locus remains heterozygous 50% of the time. One selfing event reduces heterozygosity by 50%, two consecutive selfings reduces heterozygosity by 75%, 3 consecutive events reduces it by 87.5%, and so on. Selfing in diploids therefore results in a *incremental loss* of genetic variability towards an equilibrium value given by *F_is_* = *S*/(2-*S*) where *S* is the selfing rate [25]. In contrast, a selfing event between two genetically-identically malaria haplotypes results in an *immediate and total loss* of all heterozygosity because the zygote is homozygous at all loci. The standard formula 2*F_is_*/(1+*F_is_*) is appropriate for the first situation but not for the second: in malaria selfing rate is equal to *F_it_* [35]. As a specific example, assume each human has exactly 3 equally-prevalent malaria clones in their serum that mate at random within the midgut. Selfing rate (*F_it_*) will be 33% while random mating means that, by definition, *F_is_* = 0. Substituting *F_is_* = 0 into the standard formula gives the erroneous estimate of selfing rate as zero. So selfing rate in the data of Razakandrainibe *et al* [2] is estimated as *F_it_*≈0.4 rather than the rate of 0.25 they obtained using the above formula.

Genetic analysis also provides *prima facia* evidence of non-random mating between clones in the mosquito, malaria parasites being more likely to mate with parasites from the same genetic clone than with parasites from other genetically-distinct clones present in the same blood meal ( *F_is_*>0 in Table 2). Our finding of a statistically-significant *F_is_* of 0.18 is consistent with previous analyses: Anderson *et al* [39] obtained highly significant *F_is_* values of 0.7 to 1.0 in a preliminary analysis, and Razakandrainibe *et al* [2] reported *F_is_*≈0.15,. This is clinically important because sexual-stage malaria parasites express a suite of molecules, such as *pfs*48/45, that are intimately involved in gamete recognition and binding. These are potential targets in the development of transmission-blocking vaccines [40] so it is important to understand their biological function. However significant *F_is_* values can also arise for more prosaic reasons than malaria mate recognition and choice. Anderson *et al* [39] noted that mixed blood meals (due to interrupted mosquito feeding) would result in oocysts derived from different humans being in the same midgut, and/or that outcrossing depression would both increase *F_i_*_s_. Their favoured explanation was that the presence of null mutations resulted in overestimates of *F_is_*; reanalysis of their data accounting for null mutations resulted in non-significant *F_is_* values of around 0.08 [39]. The presence of nulls is also a potential explanation for significant *F_is_* values in our analysis. Table 2 shows that significant *F_is_* values only occurred at loci where oocyst amplification had been a problem (TA109 and MSP1) implying that nulls might be present. Analysis of the two loci that reliably amplified oocysts, *TA1* and *ARA2*, both returned estimates of *F_is_* that were not significantly different from zero.

*MSP1* is a vaccine candidate and the data allow us to attempt to infer the effects of immune selection acting against the products of this locus. The principle is simple: if humans acquire immunity to the different classes of MSP1 molecules then genetic diversity within people (and hence the mosquitoes that fed on them) will decline [28]. For example, people immune to MSP1 class mad20 can only be infected by parasites expressing types k1 and ro33 and, as they subsequently also acquire immunity against k1, they can only be infected by malaria parasites expressing type ro33. Immune selection can therefore be inferred by reduced genetic variation (i.e. increased *F_st_*) in people/mosquitoes at the MSP1 locus compared to neutral loci such as microsatellite [28]. This was indeed the case (Table 2) suggestive of immune selection but the results need to be interpreted with extreme caution. Firstly, because the difference did not reach statistical significance (although previous calculations [28] suggest our sample size lacked statistical power). Secondly because problems with amplifying the MSP1 suggest the null mutations may be present which will artificially increase *F_st_* at that locus in a manner that cannot be separated from the actions of immune selection.

Malaria clones within humans may be genetically related, presumably by being co-inoculated during the same mosquito bite which can potentially inoculate hundreds of genetically-related sporozoites [41], [42]. The results of the HWE analysis for clonal relatedness were inconclusive. The data from TA109 are suspicious given the evidence, see above, that nulls were present. TA1 data suggested clones were related, ARA2 data suggested they were not. It is not clear which is correct. It may be that clones are truly related and that the results from ARA2 were a statistical type II error (failure to reject a false null hypothesis), possibly due to the rather small sample size of 40 mosquitoes with outcrossed oocysts. Alternately, it may be that the clones are truly unrelated, and that undetected nulls at the TA1 locus raised homozygosity levels; this would be indistinguishable from the effects of genetic relatedness. Paul *et al* [34] detected increased homozygosity in mixed blood infections in an analysis analogous to ours, and interpreted this as clonal relatedness although warning that “such analysis are subject to genotyping errors” (i.e. nulls). Relatedness does seems more plausible in areas of lower transmission, such as their study site, because people are more susceptible to malaria and the chances of being infected by more than one clone in a bite are presumably higher than in higher transmission sites, such as ours, where people are more immune and less susceptible. Unrelatedness between clones has been a key assumption in much modelling of drug resistance (e.g. [43]–[45]) and in data analysis (e.g. [35], [46]) and it is an important question that needs to be definitively proved or disproved, presumably by analysis of a larger dataset and explicitly considering the threat posed by the presence of nulls.

In short the data show that *P. falciparum* is undergoing genetic recombination in approximately 50% of transmissions and shows no signs of LD between loci in the major, haploid phase of its lifecycle. This conclusion is in direct contrast to one reached recently by Razakandrainibe *et al* [2] who analysed a similar dataset but concluded that their results were “consistent” with the “reproduction of genomes as clones, without recombination between gene loci” and that *P. falciparum* population structure was “clonal”, an assertion based mainly on their observation of highly significant genotypic LD values. However, they appear to have included all oocysts in their analysis, irrespective of whether they were selfed or outcrossed, and this is highly problematic. Genotypic LD, as the name suggests, searches for associations between genotypes at different loci within the diploid genotypes. *P. falciparum* is haploid in the human host so selfed oocysts must inevitably be homozygous at all loci: the results for genotypic LD therefore reflect a observation that will inevitably arise as a consequence of selfing i.e. that genotypes in selfed oocysts will consist of homozygotes associated with homozygotes at all other loci in that oocyst and, consequently, that large scale genotypic LD will be observed. This is a variation of the Bennett-Binet effect first identified 50 years ago [47]; it indicates that self-fertilisation is occurring at some unspecified rate, but it certainly does not imply that the population is essentially asexual or clonal. Razakandrainibe *et al* [2] appear to have made this extrapolation, and this difference in analysis and interpretation primarily explains why we came to such radically different conclusions about malaria sexuality. Selfed oocysts will be homozygous at all loci, but there seems no reason why the type of alleles (rather than their homozygosity) should become associated by selfing. This is why selfed oocysts were analysed separately from outcrossed oocysts, investigating the former for haplotypic LD and the latter for genotypic LD; neither showed any evidence of LD. The haploid forms of *P. falciparum* constitute the vast majority of its lifecycle and, critically, it is the haploid forms that encounter human interventions such as drug therapy and vaccine-induced immunity so it is the absence of LD in the haplotypes that is critical.

No significant LD exists in our dataset so this analysis of the diploid, sexual stages of the lifecycle produced results consistent with those obtained in the haploid, asexual stages where haplotypic LD in high transmission areas is either absent [5], [7] or present at very low levels [5], [48], the latter being explicably by transient spatial/temporal clustering of haplotypes in the dataset [5]. Most analyses are consistent is revealing sexual recombination in around 50% of oocysts (Table 3) although it is vital to note that all studies were performed in relatively high-transmission areas. In essence, the debate about malaria clonality/sexuality although longstanding and controversial (see introduction) is somewhat futile and akin to arguing about whether a glass is half full or half empty, the arguments being different ways of interpreting the same observation . The extrapolation of the results obtained in relatively high transmission areas (Table 3) to ‘low transmission’ areas not straightforward. The simplest prediction would be that the frequency of mixed infection would be proportionately lower so that the selfing rate would consequently increase. However, malaria is an extremely localised, focal disease so although average transmission rates may be low, the epidemiology may consist of patches of intense transmission (with high outcrossing rates) localised within large regions of little or no transmission (reviewed previously in [49]) so the relationship between transmission intensity and outcrossing rate is likely to be non-linear. There is some evidence for this, molecular analyses suggesting that many infected people in ‘low transmission’ areas have multiple malaria clones in their serum (and hence likely to have high outcrossing rates) [5] . Unfortunately the extremely low frequency of infected mosquito in such areas means that collecting and dissecting sufficient mosquitoes to allow direct measurement of outcrossing in infected midguts would be prohibitive. In summary, it seems inevitable that outcrossing rates will be lower in areas of low transmission, but by how much is matter for conjecture and debate. We do note in passing that even a small amount of sexual recombination has many of the advantages of obligate sexual recombination [50] with few of the costs, Sewell Wright noting that “uniparental reproduction with occasional cross-breeding gives results with favourable properties of both systems (clonal vs. sexual)”[51]. Recombination also makes it extremely difficult to maintain malaria ‘strains’ that differ in characteristics such as virulence or immune profile because the recombinants must be eliminated, placing a heavy recombination ‘load’ on the population to maintain strain ‘purity’ [10].

**Table 3:** A summary of the key results from the four published genetic analyses of oocysts i.e. from Tanzania [35], Papua new Guinea (PNG) [16], [39], Kenya [2] and Malawi (this study).

	Selfing rate	Random mating in midgut	Genotypic LD (overall)	Genotypic LD (outcrossed oocysts)	Haplotypic LD (selfed oocysts)	Clonal relatedness (within people)
Tanzania	0.33	n/a*	n/a	n/a	n/a	n/a
PNG	0.5	Yes (a/c* nulls)	n/a	n/a	n/a	n/a
Kenya	0.4	No	Yes	n/a	n/a	n/a
Malawi	0.5	Yes (a/c nulls)	Yes	No	No	inconclusive

* n/a indicates ‘not attempted’; a/c indicates ‘accounting for’.

The presence and extent of outcrossing in *P. falciparum* is a vital parameter in determining how it evolves in response to external human interventions such as novel drug and vaccine deployment. New drug therapies will be deployed as mixtures of two or more constituent drugs, and vaccines will contain mixtures of several distinct antigens. Both strategies have been deliberately adopted to ensure that mutations are required at two more genetic loci to encode drug resistance or vaccine insensitivity, thereby slowing the emergence and spread of resistance/insensitivity. Once deployed, the drugs and/or vaccines constitute a selection pressure that builds up LD between resistance-encoding loci in the parasite [43]. Sexual recombination through outcrossing breaks down these gene combinations, reducing LD, and dramatically slowing the rate at which resistance evolves [43], [44], [52]. Recombination can also interact with malaria intrahost dynamics leading to drug resistance emerging slower in areas of intermediate transmission intensity [53], [54] as well as leading to more complex population genetic dynamics [55]. Understanding the population genetics of *P. falciparum*, one of the deadliest human pathogens, is therefore clearly a research priority. This analysis of the sexual stages of *P. falciparum*, obtained in an area of moderate to high transmission of sub-Saharan Africa where most malaria mortality occurs, is the most detailed so far attempted (Table 3): it clearly show *P. falciparum* to be a sexual organism whose genomes are in linkage equilibrium; this acts to slow the emergence of drug resistance and vaccine insensitivity, extending the likely useful therapeutic lifespan of these interventions.
